# Early psychometric characteristics of the NUrsing Behavioral Engagement (NuBE) Scale in cancer settings: A three-phases validation study

**DOI:** 10.1371/journal.pone.0342693

**Published:** 2026-02-19

**Authors:** Loris Bonetti, Angela Tolotti, Andrea Bonanomi, Dario Valcarenghi, Davide Sari, Sarah Jayne Liptrott, Serena Barello

**Affiliations:** 1 Nursing Research Competence Centre, Department of Nursing, Ente Ospedaliero Cantonale (EOC), Bellinzona, Switzerland; 2 Nursing Development and Research Unit, Oncology Institute of Southern Switzerland, Ente Ospedaliero Cantonale (EOC), Bellinzona, Switzerland; 3 Department of Statistical Science, Università Cattolica del Sacro Cuore, Milan, Italy; 4 Nursing direction, Department of Nursing, Oncology Institute of Southern Switzerland, Ente Ospedaliero Cantonale (EOC), Bellinzona, Switzerland; 5 Department of Brain and Behavioural Sciences, University of Pavia, Pavia, Italy; 6 Applied Psychology Unit, IRCCS Fondazione Mondino, Pavia, Italy; Khoy University of Medical Sciences, IRAN, ISLAMIC REPUBLIC OF

## Abstract

**Background:**

Patient engagement in cancer care is increasingly recognized as essential for improving clinical outcomes. Nurses play a crucial role in fostering patient engagement, yet there is a lack of validated instruments to assess which nursing behaviors are most effective in promoting engagement from the patient’s perspective.

**Objective:**

This study aimed to develop and test the psychometric characteristics of the Nurses’ Behavioral Engagement Scale, a tool designed to measure nursing behaviors that support patient engagement in oncology settings, from patients’ point of view.

**Methods:**

The study followed a three-phase exploratory sequential mixed-methods design. In the first phase, 53 items were generated based on findings from a previous qualitative study and a systematic review of the literature. In the second phase, the items underwent content validation through a modified e-Delphi procedure with 19 experts in oncology nursing, patient engagement, tool development, and linguistics. Forty-eight items were deemed relevant and tested in the third phase with a sample of 250 cancer patients to evaluate construct validity, internal consistency, and convergent validity.

**Results:**

Exploratory Factor Analysis revealed a four-factor structure, collectively explaining 70.0% of the total variance: Factor 1 – Acknowledgment of Patient’s Uniqueness, Factor 2 – Meeting Patient’s Knowledge Expectations, Factor 3 – Fostering Patient’s Motivation, and Factor 4 – Valuing Patient’s Informal Caregivers. The final Nurses’ Behavioral Engagement Scale comprises 29 items. Internal consistency was excellent, with Cronbach’s alpha coefficients of 0.953, 0.890, 0.942, and 0.920 for the four factors, respectively. The Nurses’ Behavioral Engagement Scale demonstrated significant and meaningful correlations with the Health Care Climate Questionnaire and items assessing satisfaction with nursing care, supporting its convergent validity.

**Conclusion:**

The Nurses’ Behavioral Engagement Scale provides a psychometrically sound measure of nursing behaviors that promote patient engagement in oncology care. This tool has the potential to inform targeted interventions and quality improvement initiatives, ultimately enhancing patient-centered care and improving health outcomes in cancer patients.

## Introduction

Cancer is a significant health challenge that requires a comprehensive and patient-centered approach to healthcare delivery [1–3]. Nurses play a crucial role in this approach, not only by administering care but also by fostering patient engagement, which is essential for improving health outcomes across the cancer care continuum [4–8].

Patient engagement is increasingly recognized as a cornerstone of effective healthcare, particularly in managing complex conditions like cancer. It refers to the active participation of patients in their own healthcare, encompassing decision-making, adherence to treatment, and self-management strategies [5,9–11].

Patient engagement can be understood as a continuum with three levels: consultation, involvement, and partnership [12,13]. It begins with information sharing, progresses to active patient participation, and culminates in shared decision-making and responsibility. This framework underscores engagement as a dynamic process shaped by individual and systemic factors [14].

Further developments in the conceptualization of patient engagement [15–18] have framed it as a dynamic process involving motivation, knowledge, and behavior. This model outlines four progressive levels. At the initial stage, patients may feel uncertain and lack understanding of their condition, often adopting a passive role in their care. As they become more informed and motivated, they begin to try out health-related behaviors, though sustaining them can remain a challenge. With continued support and growing confidence, patients increasingly take an active role, making informed choices and assuming greater responsibility for their health. At the most advanced stage, health management becomes an integral part of daily life, with patients navigating the healthcare system proactively and independently. This framework highlights the interplay between personal motivation and external support in fostering meaningful patient engagement [15–18].

Some authors [19] highlight the emotional dimension of patient engagement, noting that psychological factors such as fear, anxiety, or frustration can hinder active participation. Conversely, feeling supported and empowered enhances a patient’s commitment to care. Emotional engagement interacts with cognitive and behavioral components, underscoring the need for a holistic approach to fostering and assessing patient participation [19,20].

Patient engagement is linked to improved adherence, clinical outcomes, and quality of life in cancer care [21–24]. Engaged patients communicate more effectively, participate in decisions, and report less psychological distress [25,26]. However, the role of healthcare professionals, especially nurses—in fostering engagement is still insufficiently investigated. Further research is needed to evaluate nursing behaviors and develop standardized tools to measure their impact on patient engagement.

While patient engagement denotes the patient’s active role in care, engagement-promoting behaviors refer to the strategies used by healthcare professionals to support this involvement. Nurses, in particular, facilitate engagement through communication, shared decision-making, and emotional support [27]. Distinguishing these concepts is crucial for developing accurate tools to measure healthcare professionals’ contributions to patient engagement [28].

Existing tools to assess engagement-promoting behaviors, such as the Patient Perceptions of Patient-Empowering Nurse Behaviors Scale (PPPNBS) [28] and the Caring Nurse-Patient Interaction Scale (CNPI) [29], capture aspects of empowerment and caring but do not fully encompass the scope of nursing practices that support patient engagement in oncology. This gap highlights the need for a validated instrument specifically designed to assess how nurses foster engagement in cancer care. Therefore, this study aimed to develop and test the psychometric characteristics of the Nurses’ Behavioral Engagement Scale, a tool designed to measure nursing behaviors that support patient engagement in oncology settings, from patients’ point of view.

## Materials and methods

### Setting

The study was conducted at a Swiss oncological hospital center, comprising two oncological wards (hematology and radiotherapy units) and four outpatient clinics. The NuBE Scale was tested with patients in both inpatient and outpatient settings.

### Design

This exploratory sequential mixed-methods study employed a three-phase design to develop the NUrsing Behavioral Engagement (NuBE) Scale, in accordance with established guidelines for instrument development [30–32]. Phase 1 involved conceptualization and item generation based on a systematic review [5] and a qualitative study with patients and nurses [7]. In Phase 2, content validity was evaluated through a modified e-Delphi process with expert input [33]. Phase 3 consisted of psychometric testing with oncology patients to assess the scale’s factor structure, reliability, and validity. The following sections detail the methodological procedures adopted to ensure the development of a psychometrically sound instrument.

#### Phase 1: NuBE Scale development.

**Item development procedure:** Phase 1 focused on the conceptualization of the NuBE Scale and the evaluation of face validity [34]. Item development was informed by two primary sources: a systematic review on nursing behaviors promoting patient engagement [5] and a qualitative study involving oncology patients and nurses [7]. The review, covering six databases (PubMed, CINAHL, Embase, Scopus, Web of Science, and Cochrane Library) from 2005 to 2021, included 24 eligible studies (RCTs, quasi-experimental, and pre-post designs in English, Spanish, French, or Italian) and was appraised using the Joanna Briggs Institute checklists. Three core dimensions—cognitive, emotional, and motivational—were identified as central to nursing behaviors fostering engagement, while a fourth dimension, related to informal caregivers, emerged from qualitative findings. The qualitative study involved semi-structured interviews with six cancer patients and two focus groups with 17 nurses from oncology wards and outpatient clinics. Sampling was purposive to ensure maximum socio-demographic variability. Patient interviews preceded the focus groups and informed their discussion guides. Data were transcribed verbatim and analyzed using Braun and Clarke’s thematic analysis approach [35], with methodological rigor ensured through Guba and Lincoln’s criteria for qualitative research trustworthiness [36]. The study was conducted between January and June 2021. Further methodological details are available in Tolotti et al. [7].

Based on the findings from Bonetti et al. [5] and themes emerging from the qualitative study [7] a preliminary pool of items was developed to capture nursing behaviors perceived by both patients and nurses as critical for promoting patient engagement in cancer care. The items were originally drafted in Italian.

**Consensualisation procedure and face validity:** Following item development, a consensualization process was conducted using the member checking technique [35,37], whereby participants from the qualitative phase reviewed the items to verify alignment with their experiences. Subsequently, face validity was assessed [34], focusing on content relevance, acceptability, and clarity. Based on feedback from patients and nurses, minor revisions were made to enhance comprehensibility and ensure the items accurately captured engagement-promoting nursing behaviors.

#### Phase 2: Nube Scale content validity – Modified e-Delphi procedure.

Content validity was assessed using a modified e-Delphi procedure [33], a structured consensus method widely used in instrument development. The panel included experts in patient engagement, questionnaire design, linguistics and oncological nursing. Three Delphi rounds were conducted between December 2022 and February 2023. Experts evaluated each item’s relevance to engagement-promoting nursing behaviors using a 4-point Likert scale (1 = totally irrelevant to 4 = totally relevant), following established guidelines [38,39]. Items rated as 3 or 4 were considered acceptable. Participants also provided suggestions to improve semantic clarity and content relevance. Revisions were integrated iteratively and re-evaluated in the second and third round [33,38,39].

**Content Validity Index (CVI) calculations:** Content validity metrics were computed for each Delphi round. The Item-Level Content Validity Index (I-CVI) represented the proportion of experts rating an item as 3 or 4. The Scale-Level CVI (S-CVI) indicated the proportion of items rated 3 or 4 across the full scale, while the Average S-CVI (S-CVI/Ave) was calculated as the mean I-CVI across all items [38,39]. Thresholds for adequacy were set at ≥0.83 for I-CVI and ≥0.90 for both S-CVI and S-CVI/Ave. In the third round, inter-rater agreement was further assessed using Cohen’s Kappa [40].

#### Phase 3. NuBE Scale piloting and psychometric testing.

The NuBE Scale developed in Phase 1 and validated for content by experts in Phase 2 was tested with patients. Before data collection, the scale was pilot tested with a subgroup of ten patients to assess readability and comprehensibility. No changes to the items were requested, and participants reported that the scale was easy to use. Data collection with patients took place from May 2023 to September 2023.

**Questionnaire structure:** The first section of the questionnaire included the NuBE Scale, in which patients were asked to rate the importance of each described nursing behavior in enhancing their engagement in disease management. Responses were recorded on a 7-point Likert scale, ranging from “not important at all” to “very important”.

The second section collected demographic information, including sex, age, level of education, type of oncological disease, presence of other chronic conditions, and frequency of hospitalizations in the past year.

To facilitate participation, we administered paper-based questionnaires, as most patients were older and had limited proficiency with digital devices.

**Instruments for assessing convergent validity:** To evaluate the convergent validity of the NuBE Scale, the questionnaire administered to patients included several validated instruments (see below). The selection of instruments for convergent validity was guided by established theoretical models of patient engagement, which conceptualize engagement as a multidimensional construct encompassing relational, cognitive, emotional, and motivational components. Each instrument was selected to capture a construct theoretically adjacent to engagement-promoting nursing behaviors, while remaining conceptually distinct from the dimensions assessed by the NuBE Scale.

**Healthcare Climate Questionnaire (HCCQ)** [41,42]: The Health Care Climate Questionnaire (HCCQ) was used to assess patients’ perceptions of the relational quality with their primary healthcare provider, serving as a proxy for patient engagement. The 14-item scale evaluates patient-caregiver interactions using a 7-point Likert scale (“totally disagree” to “totally agree”). Although an officially validated Italian version of the Health Care Climate Questionnaire (HCCQ) has not yet been published, the instrument has been translated into Italian and successfully used in previous studies conducted with Italian samples. For instance, Graffigna et al. [43] and Barello et al. [44] adopted the Italian-translated version of the HCCQ to assess perceived autonomy support in healthcare settings, reporting excellent internal consistency and confirming the unidimensional structure of the scale. These findings support the use of the instrument in the Italian context, even in the absence of a formally validated version.

The Cronbach’s alpha for this instrument in our study was 0.771, indicating good internal consistency.

**Patient Health Engagement Scale (PHE-S)** [45]: The PHE-S measures patients’ engagement in their healthcare journey, based on the Patient Health Engagement Model. This model conceptualizes engagement as a psychosocial process that progresses from disempowerment and vulnerability to full proactive involvement in healthcare. The Patient Health Engagement Scale (PHE-S) has been originally developed and validated in the Italian context, showing solid psychometric properties [45].

The scale consists of five self-reported items, each rated on a 7-point Likert scale (1 = low engagement; 7 = high engagement). Lower scores indicate a passive or emotionally distressed approach to healthcare, whereas higher scores reflect active and empowered health management. The total score is obtained by averaging all responses, providing an overall measure of patient engagement (Cronbach’s alpha = 0.863).

**First Question of the SF-12** [46,47]: To assess quality of life, we included the first question from the SF-12 questionnaire: “In general, would you say your health is…?”. We used the Italian version of SF-12 [46,47]. Patients responded using a 5-point Likert scale, ranging from “Excellent” to “Very Poor”. This item was included as a global self-rated health indicator rather than as a comprehensive measure of quality of life. The use of single-item self-rated health measures is well established in the literature [48] and widely adopted in health research as a valid proxy for overall health perception. In the present study, this item was included to explore associations between engagement-promoting nursing behaviors and patients’ overall health perception, rather than to capture the multidimensional construct of quality of life.

**Newcastle Satisfaction with Nursing Scale (NSNS) – Selected Items** [49,50]: To evaluate satisfaction with nursing care, we selected two general items (QN1 and QN2) from the Newcastle Satisfaction with Nursing Scale (NSNS) in Italian version [49,50]. Given the length of the full questionnaire, we focused on these two key questions, which assess:

1. The overall quality of the patient’s clinical journey
2. The quality of nursing care received

These items were selected because they capture global evaluations of the care experience and satisfaction with nursing care, which are theoretically aligned with engagement-promoting nursing behaviors. The Newcastle Satisfaction with Nursing Scale has been validated in the Italian context [49,50], and the selected items reflect high-level satisfaction judgments rather than specific procedural aspects of care.

Patients rated these items on a 7-point Likert scale, ranging from “Very Bad” to “Excellent.”

**Adherence to Refills and Medications Scale (ARMS)** [51]: The ARMS was used to measure therapeutic adherence. This scale consists of 12 questions assessing how often patients forget or intentionally skip aspects of their treatment regimen. Example items include:

- *How often do you forget to take your medicine?*
- *How often do you decide not to take your medicine?*
- *How often do you forget to get prescriptions filled?*

Responses are recorded on a 4-point Likert scale, ranging from “None” to “All.” The Adherence to Refills and Medications Scale (ARMS), although not formally validated in Italian, has been previously used in studies involving Italian patient populations, supporting its applicability in this context [52,53].

### Sampling method, inclusion and exclusion criteria and data collection procedure

To assess the early psychometric properties of the NuBE Scale, we used a convenience sample.

#### Inclusion and exclusion criteria.

Patients were eligible for inclusion if they:

- Were 18 years or older
- Were proficient in Italian
- Were able to complete a paper-based questionnaire

There were no restrictions regarding the oncological disease. Only patients receiving palliative care or those with cognitive or mental health issues, that could interfere with their ability to complete the questionnaire, were excluded.

#### Recruitment and data collection.

Patient recruitment was conducted by five nurses working in inpatient wards and outpatient clinics during the data collection period. All nurses received training on data collection procedures from the first and second authors, who also supervised the entire process to ensure consistency and adherence to the protocol.

To ensure a balanced representation of patients from different clinical contexts, we predefined the number of questionnaires for each ward and outpatient clinic. This allocation was based on the size of each unit and the patient turnover rate, ensuring proportional sampling across different settings.

#### Sample size.

According to Tabachnick et al. [54] and Watson [55] a minimum of 200 participants is recommended for Principal Component Analysis (PCA) and Exploratory Factor Analysis (EFA). Additionally, a ratio of five participants per item is generally considered sufficient.

Gorsuch [56] and Heckler [57] suggest a minimum subject-to-item ratio of 5:1 for EFA, but they emphasize that this ratio is only acceptable under specific conditions. Both authors note that higher ratios are generally preferable for more robust factor analysis. Gorsuch [56] further specifies that the 5:1 ratio is appropriate only when variable communalities are high, meaning that the selected variables share a substantial amount of variance with others and that each factor has multiple highly loaded variables. Our preliminary analyses confirmed that these conditions were met.

Based on these recommendations and considering that the initial version of the NuBE Scale consisted of 48 items, we selected a sample size of 250 participants to ensure adequate statistical power for psychometric testing.

### Statistical analysis

#### Descriptive statistics.

To describe the sample characteristics, we calculated frequencies and percentages for categorical variables and means with standard deviations for continuous variables. Moreover, we performed an item-level analysis to assess the quality of each item. Descriptive statistics were computed.

### Structural and construct validity

#### Exploratory factor analysis.

The objective was to develop a refined scale with fewer items than those identified in the e-Delphi procedure, ensuring that each factor was supported by an adequate number of items to allow for stable estimation and reliable interpretation. In line with common psychometric recommendations, a minimum of approximately five items per factor was considered a desirable guideline rather than a rigid criterion, as factors defined by a larger number of well-loading items are generally more stable and reliable, particularly in exploratory analyses [32,58,59].

Nevertheless, decisions regarding factor retention were ultimately based on a combination of statistical criteria, conceptual coherence, and interpretability, rather than on item count alone. So, scale refinement was guided by the identification of factors supported by a sufficient number of items to ensure stability and interpretability, with salient factor loadings (≥.40) and satisfactory internal consistency, as reflected by high reliability indices.

Given the exploratory nature of the study, multiple exploratory factor analyses (EFA) with an oblique Promax rotation were conducted using the principal axis factoring extraction method [59,60] which is appropriate when the goal is to identify underlying latent constructs and when the data may not meet the assumption of multivariate normality [58].

The number of factors was determined using the eigenvalue > 1 criterion [61] At each step, items were eliminated if they:

- Had low factor loadings (< 0.4), consistent with commonly adopted interpretative thresholds [59,62]
- Showed poor correlations with other items
- Had high cross-loadings on multiple factors, defined as secondary loadings ≥ .30 or differences between primary and secondary loadings smaller than.20, indicating insufficient factorial discrimination [54,59]
- Exhibited inconsistencies in shape indices

Additionally, factors were reduced if they did not have at least three or four items with significant loadings.

The appropriateness of EFA was assessed using:

- Kaiser–Meyer–Olkin (KMO) measure (considered excellent if > 0.90) [63]
- Bartlett’s test of sphericity (to confirm factorability of the correlation matrix)
- Explained variance, factor loadings, and eigenvalue >1 to define the final factorial structure

#### Reliability analysis.

Internal consistency reliability was determined using Cronbach’s alpha, with the following thresholds:

-  ≥ 0.70 = Acceptable
-  ≥ 0.80 = Good
-  ≥ 0.90 = Excellent [64]

Descriptive statistics were computed for each item in the final factor solution and for the overall factor scores. To further assess scale adequacy and conduct item analysis, we calculated:

- Item-total correlations
- Cronbach’s alpha if item deleted

#### Convergent validity.

To evaluate convergent validity, factor scores were correlated with scores from:

- Healthcare Climate Questionnaire (HCCQ) [41,42]
- Patient Health Engagement Scale (PHE-S) [45]
- First question of the SF-12 scale (for quality of life) [46,47]
- Two items from the Newcastle Satisfaction with Nursing Scale (NSNS) [49,50] (assessing satisfaction with nursing care)

Pearson correlation coefficients were computed, with a significance level (α) of 0.05.

### Ethical considerations

The study adhered to the Declaration of Helsinki and applicable local regulations for research involving human participants. Ethical approval was obtained from the regional ethics committee (Protocol No. 2020−00435/ CE 3595). All participants provided written informed consent, and anonymity and data privacy were ensured.

### Inclusivity in global research

Additional information regarding the ethical, cultural, and scientific considerations specific to inclusivity in global research is included in the Supplementary materials.

## Results

### Study samples’ characteristics

The study was conducted in three sequential phases, each involving distinct participant groups. Phase 1 included 6 cancer patients who participated in semi-structured interviews and 17 oncology nurses who took part in two focus groups, selected through purposive sampling to ensure maximum variability. Phase 2 involved 19 experts in oncology nursing, psychometrics, patient engagement, and linguistics who participated in a three-round modified e-Delphi process to assess content validity. In Phase 3, a convenience sample of 250 oncology patients was recruited from inpatient and outpatient units of a Swiss oncological hospital center to evaluate the psychometric properties of the NuBE Scale. Eligibility criteria for patients included being 18 years or older, proficient in Italian, and able to complete a paper-based questionnaire. Patients receiving palliative care or presenting cognitive or psychiatric impairments were excluded. A summary of sample characteristics for each phase is provided in Table 1, describing an overview of the sample composition, settings, and methodological purposes that informed the development and validation of the NuBE Scale. Further details related to sampling procedures and data collection are reported within the sections dedicated to each respective phase.

**Table 1:** Sample characteristics by research phase.

Phase	Participants	Setting	Purpose	Demographic Details
Phase 1 – Qualitative Study	6 patients (semi-structured interviews); 17 nurses (2 focus groups)	Oncology wards and outpatient clinics	Item generation based on qualitative insights	Purposeful sampling for maximum variability; data saturation used to define sample size
Phase 2 – e-Delphi Procedure	19 experts: 15 oncology nurses, 1 psychometrician, 2 patient engagement experts, 1 linguist	Remote (email-based Delphi rounds)	Content validation of the NuBE Scale items	Not applicable (expert panel)
Phase 3 – Psychometric Testing	250 oncology patients	Swiss oncological hospital center (inpatient and outpatient units)	Evaluation of factor structure, internal consistency, and validity	58.8% female; Mean age = 63.2 years (SD = 14.3); 29.2% with additional chronic conditions

### Phase 1: NuBE Scale development

The initial version of the NuBE checklist comprised 53 items, developed from eight core dimensions identified through a qualitative study with oncology patients and nurses [7] and further substantiated by existing literature, particularly the systematic review by Bonetti et al. [5], which highlighted cognitive, emotional, and motivational domains as central to fostering patient engagement.

The eight dimensions were as follows:

1. **Providing information and involving patients in decision-making** – Emphasizing the need for clear and accessible information to support informed participation [5]
2. **Acknowledging and respecting patient individuality** – Highlighting the importance of personalized care in building trust and engagement [7]
3. **Offering advice on therapy and lifestyle modifications** – Identified as essential for promoting self-management and treatment adherence [5,7]
4. **Stimulating and sustaining patient motivation** – Recognized as a key nursing behavior for long-term engagement [5,7]
5. **Establishing a trusted relationship** – Trust in the nurse-patient relationship was reported as critical for enabling engagement [5,7]
6. **Addressing and validating patient emotions** – Emotional support was found to reinforce commitment to care [5,7,45]
7. **Serving as a reliable point of reference** – Consistency and accessibility of nursing support enhanced patients’ sense of security [5,7]
8. **Involving family and caregivers** – Family engagement was seen as instrumental in supporting motivation and adherence [7]

For each dimension, 6–7 items were drafted using the original language of interview and focus group participants to maintain authenticity and enhance content validity. This integrative approach ensured that the NuBE Scale captures both patient and professional perspectives and is aligned with established models of engagement in oncology nursing.

#### Consensualisation procedure and face validity.

Face validity was assessed with six patients and six nurses from the qualitative phase. Participants confirmed that the items accurately reflected their experiences and were appropriate for capturing engagement-promoting nursing behaviors. Only minor revisions were made to improve clarity and comprehensibility.

### Phase 2: NuBE Scale content validity – Modified e-Delphi procedure

Nineteen experts participated in the e-Delphi process, including 15 oncology nurses, 1 psychometrician, 2 specialists in patient engagement, and 1 linguist with expertise in psychometric item development.

A total of three rounds were conducted to finalize the NuBE Scale for testing with patients. In the first, second, and third Delphi rounds, feedback was provided by 14, 12, and 15 out of the 19 experts, respectively.

- First round: Five out of fifty-three items were removed, as they received an Item-CVI score below 0.50 (i.e., fewer than 50% of experts rated them as 3 or 4). Additionally, thirty-two items were modified based on expert feedback.
- Second round: CVI scores remained below the acceptable threshold (Scale-CVI = 0.77; Average-CVI = 0.87), with five items scoring below 0.75 on the Item-CVI, which were further revised.
- Third and final round: The CVI scores reached acceptable levels (Scale-CVI = 0.94; Average-CVI = 0.92). Kappa coefficient at the third Delphi round had an average value of 0.754 (the single values range from 0,375 to 0.978), which is considered substantial agreement according to Landis & Koch [40].

**Fig 1:**
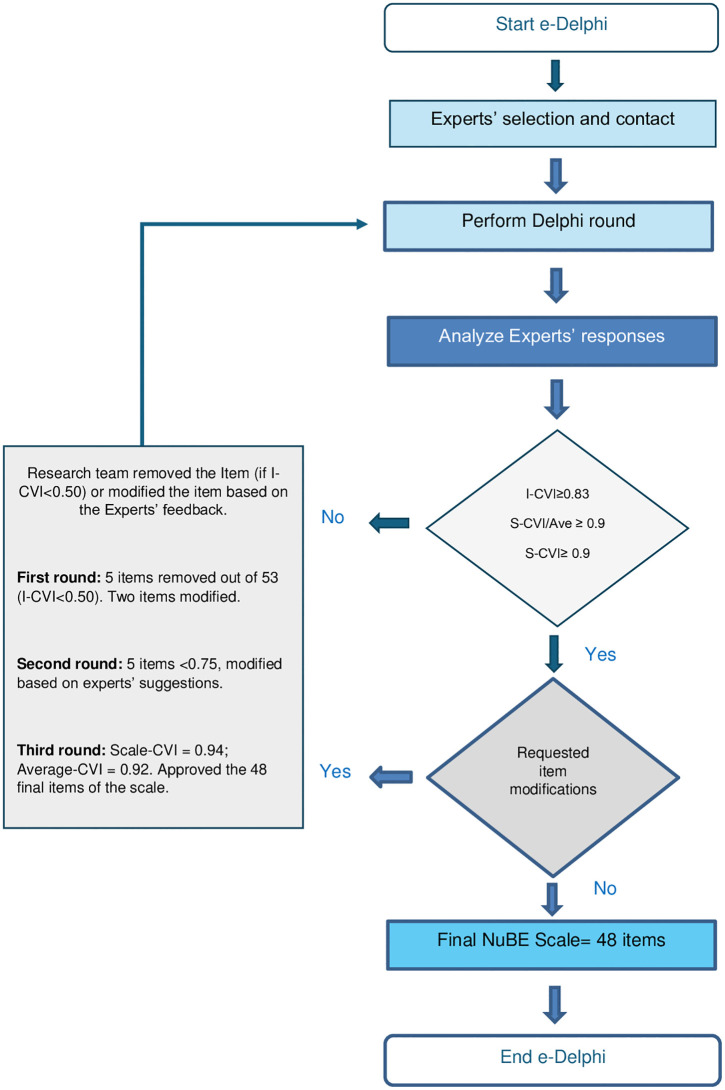
e-Delphi flow diagram.

Following the Delphi process, the NuBE Scale consisted of forty-eight items across eight dimensions. The fifty-three items tested in the Delphi rounds, along with modifications, and the final items tested with patients are included in Supplementary Materials. Fig 1 presents a flow diagram illustrating the e-Delphi rounds.

### Phase 3. NuBE Scale psychometric testing

#### Sample’s description.

A total of 250 oncology patients participated in the study (58.8% female; age range: 16–88 years; M = 63.2, SD = 14.3). All had a confirmed cancer diagnosis, and 29.2% reported additional chronic conditions. Overall, 44% had experienced at least one hospitalization in the previous year (M = 2.3). Educational attainment varied, with the majority having completed secondary (16.4%) or high secondary education (49.6%). Further socio-demographic details are provided in Table 2.

**Table 2:** Characteristics of participants (n = 250).

	*mean (SD)/ n (%)*
Age	63.2 (14.3)
Sex	
Male	102 (40.8%)
Female	147 (58.8%)
Prefer not to declare	1 (.4%)
Education	
Elementary school	14 (5.6%)
First secondary school	40 (16%)
High secondary school	124 (49.6%)
Degree	43 (17.2%)
Doctorate	5 (2.0%)
Not declared	24 (9.6%)
Other chronic illnesses in addition to cancer	
Yes	73 (29.2%)
No	177 (70.8%)

#### Psychometric properties on the NuBE scale.

**Structural and construct validity:** The aim was to develop a concise yet psychometrically robust scale; the initial version included 48 items. Descriptive statistics for all items are reported in Supplementary materials. To refine the scale and optimize its psychometric structure, successive exploratory factor analyses (EFA) with Promax rotation were conducted on the complete dataset (n = 250; no missing data). Items were excluded based on low factor loadings (<.40), substantial cross-loadings, or communalities below.20. A strong inter-item correlation structure was confirmed, and no outliers were identified. The final EFA yielded a four-factor solution comprising 29 items (Supplementary Materials), confirmed also by the Scree Plot (Supplementary material), following the procedure proposed by Cattell [65], with significant inter-factor correlations supporting the use of oblique rotation. Sampling adequacy was excellent (KMO = 0.925; Bartlett’s test: χ² = 7184, df = 406, p < .001). The four factors explained 70.0% of the total variance, with eigenvalues ranging from 6.1 to 3.6 and individual contributions of 20.9%, 18.5%, 18.2%, and 12.5%, respectively. Full factor loadings, communalities, and reliability indices are provided in Table 3.

**Table 3:** 4-Factor final solution (EFA).

Items	Factor 1 Acknowledgement of patient’s uniqueness	Factor 2 Meeting patient’s knowledge expectations	Factor 3 Fostering patient’s motivation	Factor 4 Valuing patient’s informal caregivers	Communality	Item-total correlation
Item 25. Ensure my emotions are understood and validated.	0.861				0.761	.845
Item 20. Ensure I feel free to express my emotions.	0.835				0.755	.838
Item 26. Make me feel accepted for who I am.	0.842				0.832	.881
Item 21. Make me feel that my voice is heard and important.	0.769				0.753	.826
Item 23. Acknowledge and take my feelings into consideration.	0.763				0.778	.847
Item 19. Make me feel valued throughout my treatment process.	0.736				0.605	.745
Item 27. Respect my time during the course of my care.	0.716				0.781	.850
Item 29. Make me feel welcomed in the care environment.	0.497				0.713	.797
Item 12. Provide me with opportunities to ask questions about my illness and its treatment.		0.853			0.66	.757
Item 34. Provide me with access to a skilled and competent team		0.845			0.689	.770
Item 14. Allow me to seek advice about the therapeutic procedures I will undergo.		0.785			0.702	.729
Item 16. Ensure I can place my trust in my caregivers.		0.785			0.604	.705
Item 41. Reassure me that there is a dedicated team behind the nurse, working on my behalf.		0.693			0.564	.670
Item 15. Enable me to request advice on managing potential complications related to my therapy.		0.580			0.649	.740
Item 45. Help me understand the reasoning behind the proposed treatments.		0.612			0.495	.611
Item 46. Provide opportunities to ask for explanations about what will happen to me.		0.546			0.397	.586
Item 40. Use language that I can easily understand.		0.518			0.649	.722
Item 39. Support me in maintaining my resolve and not giving up.			0.934		0.863	.884
Item 10. Encourage me to maintain a positive attitude.			0.893		0.69	.798
Item 38. Motivate me to take action and respond positively to challenges.			0.867		0.851	.884
Item 11. Support me in taking an active role in managing my illness, including its symptoms and treatments.			0.674		0.557	.733
Item 28. Instill confidence in my ability to cope with my condition.			0.676		0.766	.822
Item 37. Encourage me to set personal goals for my care and recovery.			0.688		0.735	.807
Item 35. Demonstrate genuine interest in my condition.			0.662		0.801	.814
Item 4. Ensure that those close to me, such as family members, are informed about my condition, if I desire.				0.995	0.84	.843
Item 3. Involve individuals important to me, such as family members, in my treatment process, if I wish.				0.976	0.83	.837
Item 9. Allow those close to me, such as family members, to be informed about my treatment pathway, if I so choose.				0.806	0.714	.800
Item 47. Facilitate informing those close to me, such as family members, about my health and treatment pathway, if I so choose.				0.781	0.749	.837
Item 48. Arrange for the presence of those close to me, such as family members, during visits, if I desire.				0.586	0.525	.680
Eigenvalues	6.059	5.364	5.267	3.616		
Total variance explained	20.895	18.497	18.163	12.470		
Cronbach’s Alpha	.953	.890	.942	.920		
Number of items	8	9	7	5		
Mean (SD)	52.7 (5.2)	53.7 (4.2)	45.3 (6.1)	31.7 (5.5)		

Psychometric analysis identified four factors representing the construct of Nursing Behavioral Engagement: (1) *Acknowledgment of Patient’s Uniqueness*, (2) *Meeting Patient’s Knowledge Expectations*, (3) *Fostering Patient’s Motivation*, and (4) *Valuing Patient’s Informal Caregivers*. Internal consistency was excellent across all factors (α = .953,.890,.941, and.920, respectively). Factor 1 comprised items 19, 20, 21, 23, 25, 26, 27, and 29; Factor 2 included items 12, 14, 15, 16, 34, 40, 41, 45, and 46; Factor 3 included items 10, 11, 28, 35, 37, 38, and 39; and Factor 4 included items 3, 4, 9, 47, and 48. The scale does not yield a total score; instead, four subscale scores are calculated by summing item responses within each factor. Higher scores reflect greater levels of the corresponding construct. Table 4 presents the inter-scale correlations, which indicate strong relationships among the factors.

**Reliability analysis:** Item-total correlations ranged from 0.586 to 0.884, indicating strong internal consistency. Cronbach’s alpha values remained stable when individual items were removed, confirming their contribution to the scale. These results support the NuBE Scale’s reliability as a measure of nursing behaviors that foster patient engagement.

**Convergent validity:** To evaluate the convergent validity of the NuBE Scale, we computed bivariate Pearson correlations between the factor scores and the scores of the two subscales and total score of the Healthcare Climate Questionnaire (HCCQ) [41,42] and the Patient Health Engagement Scale (PHE-S) [45]. Additionally, correlations were examined with the first question of the SF-12 [46,47], which assesses quality of life, and with two items of the Newcastle scale measuring satisfaction with nursing care received [49,50]. Convergent validity was examined by testing theory-driven hypotheses regarding the expected associations between NuBE factors and conceptually related constructs.

The analysis aimed to test specific hypotheses regarding the relationships between the NuBE Scale and other validated instruments. In particular, it was hypothesized that the first two dimensions of the NuBE Scale, Acknowledgment of Patient’s Uniqueness and Meeting Patient’s Knowledge Expectations, would show significant correlations with the HCCQ [41,42], as both constructs relate to the quality of patient-provider interactions and the level of autonomy support provided by healthcare professionals. Similarly, the PHE-S Scale [45], which measures patient engagement as a psychological process, was expected to correlate with the third factor of the NuBE Scale, Fostering Patient’s Motivation, since both constructs emphasize the role of emotional and motivational support in enhancing patient involvement.

Furthermore, it was expected that the two ad hoc items assessing satisfaction with nursing care received would correlate with all factors of the NuBE Scale, as the scale captures key nursing behaviors that contribute to patient satisfaction. However, no specific hypothesis was formulated regarding the first item of the SF-12 Scale [46,47], which assesses general health condition, as this measure does not directly align with the dimensions of the NuBE Scale.

The results of these correlation analyses are presented in Table 4, providing further insights into the relationships between the NuBE Scale and established measures of patient engagement, satisfaction, and perceived quality of care.

**Table 4:** Pearson correlation between NuBE Scale and HCCQ, PHE-S, SF12, and NSNS item ad hoc.

	NuBE F1	NuBE F2	NuBE F3	NuBE F4	HCCQ	PHE-S	SF12	QNSNS1	QNSNS2
NuBE F1	–								
NuBE F2	.757**	–							
NuBE F3	.723**	.629**	–						
NuBE F4	.481**	.417**	.461**	–					
HCCQ	.368**	.374**	.344**	.295**	–				
PHE-S	.019	.083	.032	−.114	.118	–			
SF12	.064	.083	.085	−.029	.034	.458**	–		
QNSNS1	.293**	.359**	.254**	.145*	.417**	.048	.133*	–	
QNSNS2	.283**	.324**	.288**	.161*	.473**	.073	.217**	.824**	–

HCCP: Healthcare Climate Questionnaire; NuBE: NUrsing Behavioral Engagement scale; PHE-S: Patient Health Engagement Scale; SF12: first item of the quality of life scale; QNSNS1 and QNSNS2: two items of the Newcastle Satisfaction with Nursing Scale; *p < .05; **p < .01.

All factors of the NuBE scale show significant and meaningful correlations with the total score of the HCCQ scale [41,42] and the two items regarding satisfaction with nursing care received [49,50] (particularly the first three factors). However, no significant correlations emerge for any factor with either the PHE-S [45] or the first item of the SF-12 [46,47]. Finally, very strong and significant correlations emerge among the four factors of the NuBE scale, ranging from.417 to.757.

## Discussion

The present study aimed to develop and validate the NuBE Scale, a novel instrument for assessing nursing behaviors that support patient engagement in oncology care. Findings indicate that the scale demonstrates robust psychometric properties, including face validity, internal consistency, and predictive validity. Specifically, NuBE scores were significantly and positively correlated with the total score of the Health Care Climate Questionnaire (HCCQ) [41,42], which measures patients’ perceptions of autonomy support, as well as with selected items evaluating satisfaction with nursing care. To the best of our knowledge, this is the first validated instrument specifically designed to assess engagement-promoting nursing behaviors from the patient’s perspective.

The NuBE Scale effectively captures key behavioral dimensions critical to fostering patient engagement, offering actionable insights for nursing practice. Its predictive validity suggests it can serve as a valuable tool for identifying behavioral domains that may require targeted improvements to optimize patient-centered care and health outcomes.

Compared to existing instruments such as the Patient Perceptions of Patient-Empowering Nurse Behaviors Scale (PPPNBS) [28] and the Caring Nurse-Patient Interaction Scale (CNPI) [29], the NuBE Scale presents several distinguishing features. Unlike these tools, it was developed through a sequential mixed-methods design that integrated empirical evidence with patient and nurse perspectives, thereby enhancing its content validity and contextual relevance for oncology settings.

The four-factor structure of the NuBE Scale aligns closely with established conceptualizations of patient engagement described in the literature [5,7,66] which conceptualize engagement as a dynamic and multidimensional process involving emotional, cognitive, motivational, and relational components that evolve over the care trajectory [15,18,19,26,67],. The first factor, *Acknowledgment of Patient’s Uniqueness*, reflects a core person-centered and emotional dimension of engagement, emphasizing recognition, validation, and respect for individual experiences, values, and emotions. This dimension is consistent with person-centered care and engagement frameworks highlighting emotional attunement, relational sensitivity, and recognition of patient individuality as essential preconditions for patients’ willingness to actively participate in care, particularly in vulnerable contexts such as oncology [7,19,20,68,69].

The second factor, *Meeting Patient’s Knowledge Expectations*, corresponds to the cognitive dimension of engagement and mirrors well-established models that identify information clarity, understanding, and sense-making as foundational mechanisms for informed participation, shared decision-making, and autonomy-supportive care [13,18,27,67,70,71].

By addressing patients’ informational needs through accessible and responsive communication, this dimension reflects nurses’ key role in enabling cognitive engagement and supporting patients’ capacity to make informed choices.

The third factor, *Fostering Patient’s Motivation*, maps onto the motivational and behavioral components of engagement emphasized in patient activation and Patient Health Engagement frameworks. This dimension captures nursing behaviors that support patients’ confidence, self-efficacy, and perseverance, facilitating the transition from passive adaptation to proactive involvement in care and self-management over time [15,16,19,72–74].

Finally, *Valuing Patient’s Informal Caregivers* extends the conceptualization of engagement beyond the individual patient to the broader relational ecosystem in which care is embedded, in line with family-centered and relational models of engagement that recognize the critical role of caregivers in sustaining motivation, emotional support, and adherence throughout the cancer trajectory [7,22,27,67]. Taken together, these four dimensions operationalize engagement-promoting nursing behaviors in a way that is theoretically coherent with contemporary engagement frameworks, while offering a patient-centered and practice-oriented translation of engagement theory into observable nursing behaviors.

The NuBE Scale holds promise as a tool for advancing the measurement and promotion of patient engagement in oncology care. Its application in clinical settings may support the evaluation and improvement of nursing practices, while also informing the development of educational programs and quality improvement initiatives. Furthermore, it offers a foundation for future research exploring the determinants and outcomes of engagement-promoting behaviors, including the role of nurses’ self-efficacy and perceived barriers to implementation.

### Strengths and limitations

Despite the strengths of this study, several limitations should be noted. The generalizability of the findings may be limited by the demographic and contextual characteristics of the sample, which consisted primarily of oncology patients recruited from a single healthcare network. Future studies should seek to validate the NuBE Scale in diverse clinical settings and populations to enhance its external validity. Additionally, although scale development involved extensive consultation with patients and healthcare professionals to ensure content relevance and comprehensiveness, further refinement may be warranted. Test–retest reliability was not assessed due to challenges in securing follow-up participation within this clinically vulnerable population. A primary limitation of this study is the absence of confirmatory factor analysis (CFA) to cross-validate the EFA-derived structure. To avoid overfitting, CFA was not conducted on the same sample due to size constraints. Future research should replicate these findings using CFA in an independent, adequately powered sample and assess the scale’s test-retest reliability. Nonetheless, this study offers a robust initial validation of the NuBE Scale and supports its psychometric potential.

An additional limitation is the absence of Content Validity Ratio (CVR) analysis, which was intentionally omitted to preserve a broad item pool during the initial evaluation phase. Future research may incorporate CVR calculations to further refine the scale and assess item relevance and clarity across varied healthcare contexts.

Additionally, the use of selected items and single-item indicators for convergent validity represents a pragmatic compromise aimed at reducing respondent burden in a clinically vulnerable population; future studies should replicate these findings using multidimensional measures to further strengthen construct validation.

## Conclusion and practical implications

The validation of the NuBE Scale represents an important step toward advancing patient-centered care by offering a psychometrically robust tool to assess nursing behaviors that promote patient engagement. By addressing the specific needs of oncology patients, the scale can inform the design of targeted interventions and quality improvement initiatives aimed at enhancing care delivery and outcomes in this population.

Practically, the NuBE Scale has broad applicability across clinical, educational, and policy contexts. Clinically, it can function as a quality assessment tool to monitor engagement-related nursing behaviors and guide tailored improvements. In nursing education, it can support training programs focused on developing engagement competencies. At the policy level, the scale provides measurable indicators for embedding engagement-oriented practices into care models. Additionally, it offers a valuable resource for research exploring the long-term effects of nursing behaviors on patient engagement and clinical outcomes.

Overall, the NuBE Scale contributes to the systematic evaluation and promotion of engagement-enhancing nursing practices in oncology and beyond.

## Supporting information

S1 File Inclusivity-in-global-research-checklist. (PDF)

S1 Data Dataset. (CSV)

S2 File Delphi rounds evaluation with experts. (PDF)

S3 File Items tested with patients. (PDF)

S4 File Item analysis. (PDF)

S5 File Final scale. (PDF)

S6 File Scree plot. (PDF)
