# Supplementary material for: Early psychometric characteristics of the NUrsing Behavioral Engagement (NuBE) Scale in cancer settings: A three-phases validation study
Source: PLoS One. 2026 Feb 19;21(2):e0342693. doi: 10.1371/journal.pone.0342693 (PMC12919838; doi:10.1371/journal.pone.0342693)
Supplement: S2 File — (PDF) [file pone.0342693.s003.pdf]

Supplementary materials 2: Items validation/adaptation during the Delphi rounds for content validity.

N	Original Item	Item revision round 1	Item revision round 2	Item revision round 3
1.	Being involved in therapy decisions	Being actively involved in the treatments offered to me.	approved	approved
2.	Being listened to	approved	approved	approved
3.	That people important to me are involved	Involve individuals important to me, such as family members, in my treatment process, if I wish.	approved	approved
4.	That people close to me know about my condition	Ensure that those close to me, such as family members, are informed about my condition, if I desire.	approved	approved
5.	To receive guidance on whom to contact in times of difficulty	Receive guidance on whom to contact during times of difficulty related to my illness.	Having the opportunity to seek guidance on whom to contact during times of difficulty related to my illness.	approved
6.	Receiving support during times of despair	approved	approved	approved
7.	Being connected with patients who have experienced a similar situation to mine.	approved	approved	approved
8.	To receive lifestyle advice	Having the opportunity to seek approved lifestyle advice.	approved	approved
9.	That people close to me know the path I will have to take	That people close to me know about the clinical course I will have to undergo	Allow those close to me, such as family members, to be informed about my clinical course, if I so choose.	approved
10.	Being encouraged to maintain a positive attitude	approved	approved	approved

11.	Being encouraged to become a protagonist	Being encouraged to take an active role in managing my disease, including its symptoms and treatments.	approved	approved
12.	Being able to ask about the disease and treatment	Having the opportunity to request information about my disease and its treatment.	approved	approved
13.	Receiving advice on how to manage my symptoms	approved	approved	approved
14.	To receive advice on my treatment procedures	Receive advice on the therapeutic procedures I will face	Having the opportunity to seek advice about the therapeutic procedures I will undergo.	approved
15.	To receive advice on how to manage possible complications	Receive advice on how to manage possible complications related to treatment for my disease	Having the opportunity to seek advice on managing potential complications related to my therapy.	approved
16.	Being able to trust	Being confident in my ability to trust my caregivers.	approved	approved
17.	Not feeling guilty	Feeling assured that I am not being blamed.	approved	approved
18.	Feeling confident that I am not being judged.	approved	approved	approved
19.	Feeling that my strengths are taken into account	Feeling appreciated and valued throughout my treatment.	approved	approved
20.	Feeling free to express my emotions	Feeling free to express my emotions.	approved	approved
21.	Feeling that my voice is heard and important.	approved	approved	approved
22.	Receiving care tailored to my personal needs.	approved	approved	approved
23.	Feeling that my feelings are acknowledged and taken into account.	approved	approved	approved

24.	Being able to express my fears	Having the opportunity to share and express my fears.	approved	approved
25.	Feeling that my emotions are understood and acknowledged.	approved	approved	approved
26.	Feeling accepted for who I am	approved	approved	approved
27.	Feeling that my time is respected.	approved	approved	approved
28.	Feeling confident in my ability to cope	approved	approved	approved
29.	Feeling welcomed and comfortable.	Approved	approved	approved
30.	Feeling that I am treated as an individual, not just a number.	Approved	approved	approved
31.	Being invited to contribute to the design of research aimed at improving therapeutic possibilities.	Not approved		
32.	Having the opportunity to support other patients in research projects.	Not approved		
33.	To receive practical examples to better understand	Having the opportunity to request practical examples to better understand my condition.	approved	approved
34.	To be followed by the same nurse	Receiving continuity of care by being followed, if possible, by the same nurse.	approved	approved
35.	Being involved in the design of research projects.	Not approved		
36.	Actively participating in oncology research.	Not approved		
37.	To be able to rely on the nurse of reference when needed	Being confident in the support of a trained and competent team.	approved	approved
38.	To know that I can count on a prepared and competent team	Being able to count on a trained and competent team	approved	approved
39.	Perceiving their interest in my condition	Perceiving genuine interest in my condition.	approved	approved
40.	Perceiving their availability	Feeling their willingness to support me.	approved	approved

41.	Being encouraged to set myself goals	Being encouraged to set personal goals.	approved	approved
42.	Feeling spurred to react in the face of difficulties	Feeling motivated to respond positively when faced with difficulties.	approved	approved
43.	Being encouraged not to give up	Being supported to maintain my resolve and not give up.	approved	approved
44.	That comprehensible language is used / used	Having information communicated in language I can easily understand.	approved	approved
45.	Being offered the opportunity to participate in research to advance knowledge about my illness.	Not approved		
46.	To know that behind the nurse there is a coordinated team caring for me	Knowing that behind the nurse, there is a team that cares for me.	approved	approved
47.	not having to repeat my path to different people all the time	Avoiding the need to repeatedly share my medical history with different caregivers.	approved	approved
48.	Being able to apologise when necessary	Receiving an apology when appropriate.	approved	approved
49.	Being able to play down	Having the opportunity to lighten the mood if it helps me feel better.	approved	approved
50.	To understand the rationale behind the therapies that are proposed to me	approved	approved	approved
51.	That it is explained to me what will happen	Being told what will happen to me	Having the opportunity to request an explanation of what will happen to me.	approved
52.	That people close to me are made aware of the situation	Allowing those close to me, such as family members, to be informed about my health situation and treatment pathway, if I so choose.	approved	approved

53.	That people close to me are present at the visits	Arranging for those close to me, such as family members, to be present at visits, if I so wish.	approved	approved
