# Supplementary material for: Early psychometric characteristics of the NUrsing Behavioral Engagement (NuBE) Scale in cancer settings: A three-phases validation study
Source: PLoS One. 2026 Feb 19;21(2):e0342693. doi: 10.1371/journal.pone.0342693 (PMC12919838; doi:10.1371/journal.pone.0342693)
Supplement: S3 File — (PDF) [file pone.0342693.s004.pdf]

Supplementary materials 3: Items tested with patients.

N	**Item tested with patients**
1.	Being involved in the treatments I am offered
2.	Being listened to.
3.	Involve individuals important to me, such as family members, in my treatment process, if I wish.
4.	Ensure that those close to me, such as family members, are informed about my condition, if I desire.
5.	Having the possibility to ask for guidance on whom to contact during times of difficulty related to my illness.
6.	Receiving support during times of despair.
7.	Being connected with patients who have experienced a similar situation.
8.	Having the opportunity to ask for approved lifestyle advice.
9.	Allow those close to me, such as family members, to be informed about my treatment pathway, if I so choose.
10.	Encourage me to maintain a positive attitude.
11.	Support me in taking an active role in managing my illness, including its symptoms and treatments.
12.	Provide me with opportunities to ask questions about my illness and its treatment.
13.	Receiving advice on how to manage my symptoms.
14.	Allow me to seek advice about the therapeutic procedures I will undergo.
15. .	Enable me to request advice on managing potential complications related to my therapy.
16.	Ensure I can place my trust in my caregivers.
17.	Make me feel that I am not being blamed.
18.	Ensure I do not feel judged.

19.	Make me feel valued throughout my treatment process.
20.	Ensure I feel free to express my emotions.
21.	Make me feel that my voice is heard and important.
22.	Provide care adapted to my personal needs.
23.	Acknowledge and take my feelings into consideration.
24.	Allow me the opportunity to express my fears.
25.	Ensure my emotions are understood and validated.
26.	Make me feel accepted for who I am.
27.	Respect my time during the course of my care.
28.	Instill confidence in my ability to cope with my condition.
29.	Make me feel welcomed in the care environment.
30.	Ensure I do not feel like just a number.
31.	Allow me to request practical examples to better understand my condition.
32.	Provide continuity of care by enabling me to be followed, if possible, by the same nurse.
33.	Ensure I can rely on a reference nurse when needed.
34.	Provide me with access to a skilled and competent team.
35.	Demonstrate genuine interest in my condition.
36.	Show willingness to support me.
37.	Encourage me to set personal goals.
38.	Motivate me to take action and respond positively to challenges.

39.	Support me in maintaining my resolve and not giving up.
40.	Use language that I can easily understand.
41.	Reassure me that there is a dedicated team behind the nurse, working on my behalf.
42.	Avoid requiring me to repeatedly share my medical history with different caregivers.
43.	Offer an apology when appropriate.
44.	Allow me the possibility to lighten the mood, if it helps me feel better.
45.	Help me understand the reasoning behind the proposed treatments.
46.	Provide opportunities to ask for explanations about what will happen to me.
47.	Facilitate informing those close to me, such as family members, about my health and treatment pathway, if I so choose.
48.	Arrange for the presence of those close to me, such as family members, during visits, if I desire.
