# Supplementary material for: Early psychometric characteristics of the NUrsing Behavioral Engagement (NuBE) Scale in cancer settings: A three-phases validation study
Source: PLoS One. 2026 Feb 19;21(2):e0342693. doi: 10.1371/journal.pone.0342693 (PMC12919838; doi:10.1371/journal.pone.0342693)
Supplement: S4 File — (PDF) [file pone.0342693.s005.pdf]

Supplementary materials 4: Item analysis

	N	Missing	Mean	Median	SD	Min	Max
**Item1**	250	0	6.569	7.000	0.975	1	7
**Item2**	250	0	6.782	7.000	0.556	4	7
**Item3**	250	0	6.336	7.000	1.271	1	7
**Item4**	250	0	6.409	7.000	1.216	1	7
**Item5**	250	0	6.543	7.000	0.960	1	7
**Item6**	250	0	6.534	7.000	0.966	1	7
**Item7**	250	0	4.664	5.000	2.208	1	7
**Item8**	250	0	5.967	7.000	1.539	1	7
**Item9**	250	0	6.350	7.000	1.215	1	7
**Item10**	250	0	6.433	7.000	1.149	1	7
**Item11**	250	0	6.392	7.000	1.164	1	7
**Item12**	250	0	6.721	7.000	0.674	3	7
**Item13**	250	0	6.688	7.000	0.724	1	7
**Item14**	250	0	6.676	7.000	0.727	2	7
**Item15**	250	0	6.671	7.000	0.794	1	7
**Item16**	250	0	6.865	7.000	0.464	4	7
**Item17**	250	0	6.490	7.000	1.133	1	7
**Item18**	250	0	6.580	7.000	0.979	1	7
**Item19**	250	0	6.504	7.000	0.865	1	7
**Item20**	250	0	6.640	7.000	0.752	3	7
**Item21**	250	0	6.610	7.000	0.763	2	7
**Item22**	250	0	6.732	7.000	0.712	1	7
**Item23**	250	0	6.632	7.000	0.769	1	7
**Item24**	250	0	6.607	7.000	0.930	1	7
**Item25**	250	0	6.547	7.000	0.784	4	7
**Item26**	250	0	6.559	7.000	0.833	1	7
**Item27**	250	0	6.537	7.000	0.764	4	7
**Item28**	250	0	6.608	7.000	0.855	1	7
**Item29**	250	0	6.672	7.000	0.670	4	7
**Item30**	250	0	6.711	7.000	0.628	4	7
**Item31**	250	0	6.304	7.000	1.186	1	7
**Item32**	250	0	5.959	7.000	1.487	1	7
**Item33**	250	0	6.370	7.000	1.127	1	7
**Item34**	250	0	6.806	7.000	0.544	4	7
**Item35**	250	0	6.528	7.000	0.906	1	7
**Item36**	250	0	6.553	7.000	0.810	1	7
**Item37**	250	0	6.335	7.000	1.102	1	7
**Item38**	250	0	6.474	7.000	0.999	1	7
**Item39**	250	0	6.492	7.000	0.966	1	7
**Item40**	250	0	6.671	7.000	0.712	2	7
**Item41**	250	0	6.728	7.000	0.628	2	7
**Item42**	250	0	6.272	7.000	1.292	1	7
**Item43**	250	0	6.020	7.000	1.416	1	7
**Item44**	250	0	6.299	7.000	1.102	1	7
**Item45**	250	0	6.634	7.000	0.878	1	7
**Item46**	250	0	6.638	7.000	0.873	1	7

**Item47**	250	0	6.362	7.000	1.193	1	7
**Item48**	250	0	6.244	7.000	1.428	1	7
