# Supplementary material for: Early psychometric characteristics of the NUrsing Behavioral Engagement (NuBE) Scale in cancer settings: A three-phases validation study
Source: PLoS One. 2026 Feb 19;21(2):e0342693. doi: 10.1371/journal.pone.0342693 (PMC12919838; doi:10.1371/journal.pone.0342693)
Supplement: S5 File — (PDF) [file pone.0342693.s006.pdf]

Supplementary materials 5: Final List of Items

N	Item	Final List of Items
1.	Item 3	Involve individuals important to me, such as family members, in my treatment process, if I wish.
2.	Item 4	Ensure that those close to me, such as family members, are informed about my condition, if I desire.
3.	Item 9	Allow those close to me, such as family members, to be informed about my treatment pathway, if I so choose.
4.	Item 10	Encourage me to maintain a positive attitude.
5.	Item 11	Support me in taking an active role in managing my illness, including its symptoms and treatments.
6.	Item 12	Provide me with opportunities to ask questions about my illness and its treatment.
7.	Item 14	Allow me to seek advice about the therapeutic procedures I will undergo.
8.	Item 15	Enable me to request advice on managing potential complications related to my therapy.
9.	Item 16	Ensure I can place my trust in my caregivers.
10.	Item 19	Make me feel valued throughout my treatment process.
11.	Item 20	Ensure I feel free to express my emotions.
12.	Item 21	Make me feel that my voice is heard and important.
13.	Item 23	Acknowledge and take my feelings into consideration.
14.	Item 25	Ensure my emotions are understood and validated.
15.	Item 26	Make me feel accepted for who I am.
16.	Item 27	Respect my time during the course of my care.
17.	Item 28	Instill confidence in my ability to cope with my condition.
18.	Item 29	Make me feel welcomed in the care environment.

19.	Item 34	Provide me with access to a skilled and competent team.
20.	Item 35	Demonstrate genuine interest in my condition.
21.	Item 37	Encourage me to set personal goals for my care and recovery.
22.	Item 38	Motivate me to take action and respond positively to challenges.
23.	Item 39	Support me in maintaining my resolve and not giving up.
24.	Item 40	Use language that I can easily understand.
25.	Item 41	Reassure me that there is a dedicated team behind the nurse, working on my behalf.
26.	Item 45	Help me understand the reasoning behind the proposed treatments.
27.	Item 46	Provide opportunities to ask for explanations about what will happen to me.
28.	Item 47	Facilitate informing those close to me, such as family members, about my health and treatment pathway, if I so choose.
29.	Item 48	Arrange for the presence of those close to me, such as family members, during visits, if I desire.

**Factor 1** Acknowledgement of patient's uniqueness	
**Factor 2** Meeting patient's knowledge expectations	
**Factor 3** Fostering patient's motivation	
**Factor 4** Valuing patient's informal caregivers	
