# Supplementary figures and images for: Early psychometric characteristics of the NUrsing Behavioral Engagement (NuBE) Scale in cancer settings: A three-phases validation study
Source: PLoS One. 2026 Feb 19;21(2):e0342693. doi: 10.1371/journal.pone.0342693 (PMC12919838; doi:10.1371/journal.pone.0342693)

Supplementary materials 6: NuBE Scree plot

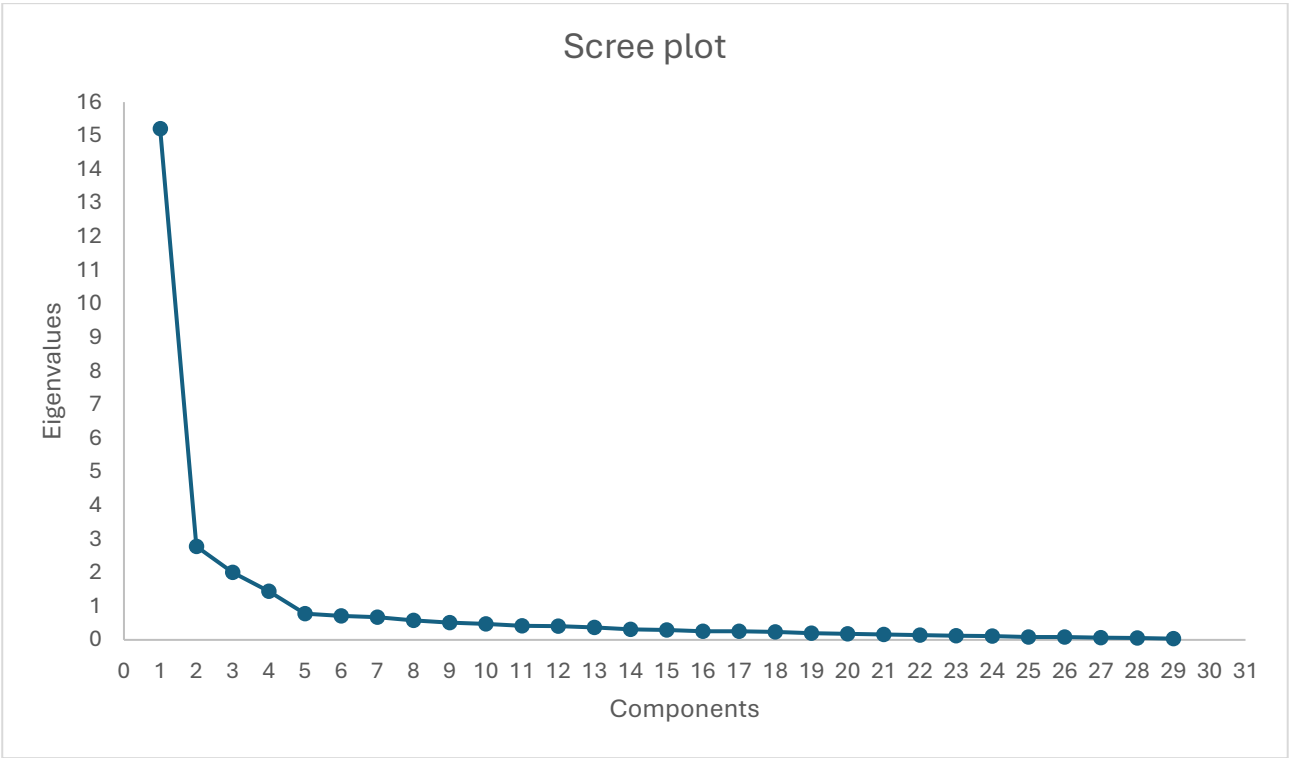

Supplement: S6 File — (PDF) [file pone.0342693.s007.pdf]
